# Type III Secretion System Genes of *Dickeya dadantii* 3937 Are Induced by Plant Phenolic Acids

**DOI:** 10.1371/journal.pone.0002973

**Published:** 2008-08-13

**Authors:** Shihui Yang, Quan Peng, Michael San Francisco, Yongjun Wang, Quan Zeng, Ching-Hong Yang

**Affiliations:** 1 Department of Biological Sciences, University of Wisconsin-Milwaukee, Milwaukee, Wisconsin, United States of America; 2 Department of Biological Sciences, Texas Tech University, Lubbock, Texas, United States of America; Centre for DNA Fingerprinting and Diagnostics, India

## Abstract

**Background:**

*Dickeya dadantii* is a broad-host range phytopathogen. *D. dadantii* 3937 (Ech3937) possesses a type III secretion system (T3SS), a major virulence factor secretion system in many Gram-negative pathogens of plants and animals. In Ech3937, the T3SS is regulated by two major regulatory pathways, HrpX/HrpY-HrpS-HrpL and GacS/GacA-*rsmB*-RsmA pathways. Although the plant apoplast environment, low pH, low temperature, and absence of complex nitrogen sources in media have been associated with the induction of T3SS genes of phytobacteria, no specific inducer has yet been identified.

**Methodology/Principal Findings:**

In this work, we identified two novel plant phenolic compounds, *o*-coumaric acid (OCA) and *t*-cinnamic acid (TCA), that induced the expression of T3SS genes *dspE* (a T3SS effector), *hrpA* (a structural protein of the T3SS pilus), and *hrpN* (a T3SS harpin) *in vitro*. Assays by qRT-PCR showed higher amounts of mRNA of *hrpL* (a T3SS alternative sigma factor) and *rsmB* (an untranslated regulatory RNA), but not *hrpS* (a σ^54^-enhancer binding protein) of Ech3937 when these two plant compounds were supplemented into minimal medium (MM). However, promoter activity assays using flow cytometry showed similar promoter activities of *hrpN* in *rsmB* mutant Ech148 grown in MM and MM supplemented with these phenolic compounds. Compared with MM alone, only slightly higher promoter activities of *hrpL* were observed in bacterial cells grown in MM supplemented with OCA/TCA.

**Conclusion/Significance:**

The induction of T3SS expression by OCA and TCA is moderated through the *rsmB*-RsmA pathway. This is the first report of plant phenolic compounds that induce the expression T3SS genes of plant pathogenic bacteria.

## Introduction

*Dickeya dadantii* (formerly *Erwinia chrysanthemi*) is an opportunistic plant pathogen that causes soft-rot, wilt, and blight diseases on a wide range of plant species. This bacterial pathogen produces a large battery of pectinases for disassembly of the plant cell wall [1]. In phytobacteria, a type III secretion system (T3SS) or hypersensitive response and pathogenicity (Hrp) system, which is responsible for the secretion and translocation of effector proteins into the host cells, is considered a major virulence factor in pathogenesis [2], [3]. Genome sequencing has revealed that *D. dadantii* 3937 (Ech3937) has a complete set of genes for the T3SS apparatus. The T3SS in *D. dadantii* has also been reported to play a role in pathogenicity [4]–[8].

**Figure 1:**
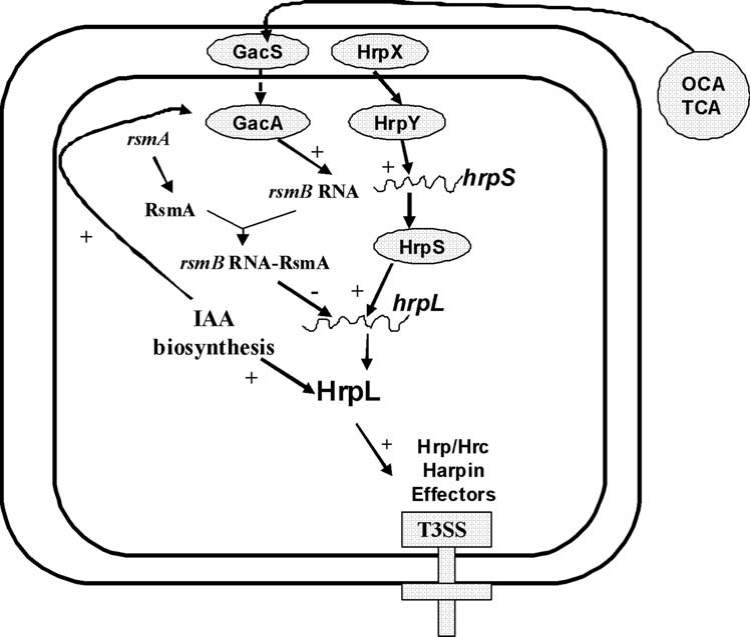
Model of plant phenolic compounds *o*-coumaric acid (OCA) and *t*-cinnamic acid (TCA) that induce expression of the type III secretion system (T3SS) genes of *Dickeya dadantii* 3937 (Ech3937). The T3SS and Gac-Rsm regulatory cascades of Ech3937 were adopted as described [15], [16], [19], [20].

The expression of T3SS genes in phytobacteria is repressed when bacterial cells are cultured in complex media, but is induced in the plant apoplast or in close contact with host cells [9]–[14]. Expression of T3SS genes is also induced in minimal medium (MM), which is considered to mimic plant apoplastic conditions [14]. The T3SS of Ech3937 is regulated by two major regulatory pathways, the HrpX/HrpY-HrpS-HrpL and the GacS/GacA-*rsmB*-RsmA pathways [15], [16]. In the HrpX/HrpY-HrpS-HrpL pathway, the HrpX/HrpY, which is a two-component system (TCS), activates the gene encoding HrpS, which is a σ^54^-enhancer binding protein (Fig. 1). The HrpS protein activates the expression of an alternative sigma factor, *hrpL*. HrpL is required for the expression of genes encoding the T3SS effectors and structural components such as the units of the needle, the needle extension, and the translocon [16]. In the GacS/GacA-*rsmB*-RsmA pathway, the RsmA protein promotes the decay of *hrpL* mRNA [15], [17]. *rsmB* is an untranslated regulatory RNA that binds RsmA and neutralizes its negative regulatory effect of RsmA by forming an inactive ribonucleoprotein complex [15], [17]–[20]. Although the signal molecule for GacS autophosphorylation is still unknown, the TCS GacS/GacA is reported to up-regulate *rsmB* [15], [17].

Although several environmental factors (e.g., low pH, low temperature, and the absence of complex nitrogen sources in media) were found to influence the expression of T3SS genes in phytobacteria, no specific plant inducer for *hrp* gene expression has been identified [12], [13], [21]–[24]. Several phenolic acids were reported to play dominant roles in defense signaling in plants [25], [26]. Recently, efflux pump genes of Ech3937 were found to be induced by phenolic acids [26], which have been suggested to be essential for the pathogenesis of the bacterium by enhancing the resistance to antimicrobial plant chemicals [27]. Since one major role of T3SS of phytopathogens is to neutralize the host defense system during bacterial invasion, it is possible that Ech3937 induces expression of T3SS genes by recognizing certain phenolic compounds in plants. To identify plant compounds that induce the expression of T3SS genes, we focused on elucidating the effect of *o*-coumaric acid (OCA), *t*-cinnamic acid (TCA), and salicylic acid (SA) on *hrp* expression and on the T3SS regulatory pathway.

In this study, two novel plant phenolic compounds, OCA and TCA, that induce the expression T3SS genes of Ech3937, is described. In addition, the regulatory effect for T3SS gene induction by these two phenolic compounds is elucidated.

## Results

### T3SS gene expression is induced by plant phenolic compounds

**Figure 2:**
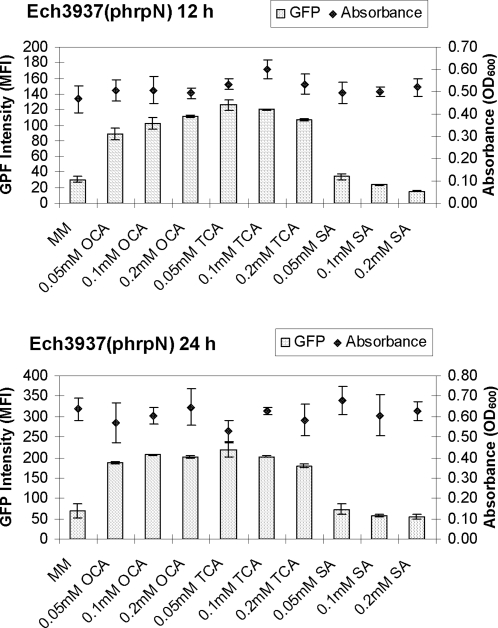
The promoter activities of *hrpN* in *Dickeya dadantii* 3937 (Ech3937) grown in MM and MM supplemented with 0.05, 0.1, and 0.2 mM OCA, TCA, and SA at 12 h and 24 h post- inoculation. GFP intensity was determined on gated populations of bacterial cells by flow cytometry and analyzed with the Cell Quest software (BD Biosciences, San Jose, CA). The growth of Ech3937 in MM supplemented with different concentrations of OCA, TCA and SA was recorded.

Our previous efforts to screen the plant up-regulated genes in Ech3937 demonstrated that *dspE* and *hrpA* were expressed *in planta* [28]. Phenolic compounds constitute an important class of organic substances produced by plants. The phenolic compound SA is a signaling molecule that plays a role in host defenses. OCA and TCA are the biosynthetic precursors of SA and are also reported to induce the expression of defense-related genes in plants [29], [30]. We examined OCA, TCA, and SA to elucidate their effect on the expression of T3SS genes. The expression of the T3SS gene *hrpN* was examined in MM and MM supplemented with OCA, TCA, and SA, at concentrations of 0.05, 0.1, and 0.2 mM, respectively. Compared with minimal *hrp*-inducing medium (MM) alone, the average GFP fluorescence intensity of bacterial cells of Ech3937 (phrpN) (Table 1) was increased approximately 4-fold when 0.05 mM of OCA and TCA were added to the medium (Fig. 2). The addition of SA did not result in increased GFP fluorescence intensity of Ech3937 (Fig. 2). No obvious inhibition of bacterial growth was observed when OCA, TCA, and SA were added into the MM at the concentration below 0.2 mM (Fig. 2; Supplementary Fig. S1).

**Table 1:** Strains, plasmids, and DNA primers used in this study.

Strains, plasmids and primers	Characters or sequences (5′ to 3′)^a^	Reference or source
**Strains**		
***E. coli***		
* E. coli* DH5α	F^−^ *φ*80*lacZΔM15 ΔlacZYA-argF*)*U169 deoR recA1 endA1 hsdR17 phoA supE44 thi-1 gyrA96 relA1 λ^−^*	Invitrogen, CA
* E. coli* TOP10	F^−^ *mcrA Δmrr-hsdRMS-mcrBC*) *φ*80*lacZΔM15 ΔlacX74 deoR recA1 araD139 Δ*(*ara-leu*)*7679 galU galK rpsL endA1 nupG*	Invitrogen, CA
***D. dadantii***		
Ech3937	Wild type, *Saintpaulia* (African violet) isolate	Hugouvieux-Cotte-Pattat, N.
Ech-Rif	Ech3937 rifampicin resistant random mutant	[15]
Ech137	*ΔgacA*::*kan* constructed from Ech-Rif; Km^R^	[15]
Ech138	*ΔiaaM*::*kan*; Km^R^	[15]
Ech148	transposon mini*Himar RB1* insertion in *rsmB*, Km^R^	This work
Ech149	transposon mini*Himar RB1* insertion in *gacS*, Km^R^	This work
Ech3937 (pAT)	Ech3937 containing pPROBE-AT	[33], [34]
Ech3937 (phrpA)	Ech3937 containing p*hrpA*; Ap^R^	This work
Ech3937 (phrpN)	Ech3937 containing p*hrpN*; Ap^R^	[32]
Ech3937 (phrpL)	Ech3937 containing p*hrpL*; Ap^R^	[32]
Ech3937 (phrpS)	Ech3937 containing p*hrpS*; Ap^R^	This work
Ech3937 (pmrp)	Ech3937 containing p*mrp*; Ap^R^	[31]
Ech-Rif (phrpA)	Ech-Rif containing p*hrpA*; Ap^R^	[15]
Ech137 (phrpA)	Ech137 containing p*hrpA*; Ap^R^ Km^R^	[15]
Ech138 (phrpN)	Ech138 containing p*hrpN*; Ap^R^ Km^R^	[32]
Ech148 (phrpN)	Ech148 containing p*hrpA*; Ap^R^ Km^R^	This work
Ech149 (phrpN)	Ech149 containing p*hrpA*; Ap^R^ Km^R^	This work
**Plasmids**		
pPROBE-AT	Promoter-probe vector, Ap^R^	[33], [34]
pCR2.1-TOPO	PCR cloning vector, Ap^R^ Km^R^	Invitrogen, CA
phrpA	pProbe-AT derivative with PCR fragment containing 412-bp *hrpA* promoter region, Ap^R^	This work
phrpN	pProbe-AT derivative with PCR fragment containing *hrpN* promoter region, Ap^R^	[32]
phrpL	pProbe-AT derivative with PCR fragment containing *hrpL* promoter region, Ap^R^	[32]
phrpS	pProbe-AT derivative with PCR fragment containing 709-bp *hrpS* promoter region, Ap^R^	This work
Pmrp	pProbe-AT derivative with PCR fragment containing *mrp* promoter region, Ap^R^	[31]
**Primers**		
phrpA_F	GTGCCGATAGCCAGTGAT	This work
phrpA_R	TGCTGCTGCGTTAGAAAG	This work
phrpS_F	CAGATTGTATTTGCGGATTG	This work
phrpS_R	CGGATTCATTGCTATTCCTTAT	This work
rplU_RTF	GCGGCAAAATCAAGGCTGAAGTCG	[32]
rplU_RTR	CGGTGGCCAGCCTGCTTACGGTAG	[32]
hrpY_RTF	CGGCGACGGGCGTAATGAA	This work
hrpY_RTR	TTTCGGCGATGGCATTGACC	This work
hrpS_RTF	TGGAAGGCGAAACCGGCACC	This work
hrpS_RTR	GCACGGCGGCGCAGTTCAC	This work
hrpL_RTF	GATGATGCTGCTGGATGCCGATGT	[32]
hrpL_RTR	TGCATCAACAGCCTGGCGGAGATA	[32]
hrpA_RTF	CAGCAATGGCAGGCATGCAG	[32]
hrpA_RTR	CTGGCCGTCGGTGATTGAGC	[32]
dspE_RTF	GATGGCGGAGCTGAAATCGTTC	[32]
dspE_RTR	CCTTGCCGGACCGCTTATCATT	[32]
rsmB_RTF	AGAGGGATCGCCAGCAAGGATTGT	This work
rsmB_RTR	CGTTTGCAGCAGTCCCGCTACC	This work
gacA_RTF	GCG CTG CCC AGG AAC GTT CT	This work
gacA_RTR	CGG CCG TGG GTG GAG TCA T	This work

a Ap^R^, ampicillin resistance; Km^R^, kanamycin resistance.

**Figure 3:**
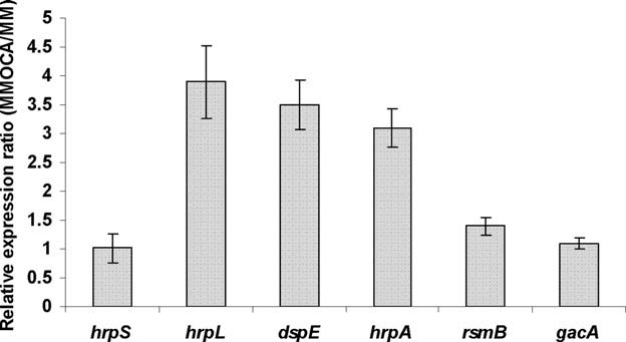
The relative mRNA level of *hrpS, hrpL, dspE, hrpA, rsmB,* and *gacA* of *Dickeya dadantii* 3937 (Ech3937) in MM supplemented with 0.1 mM OCA compared to those in MM without OCA. The amount of mRNA was determined by qRT-PCR. Three replicates were used in this experiment. The *p*-value was calculated using Relative Expression Software Tool as described by Pfaffl et al. [41]. There is no significant difference between MM and MM supplemented with OCA for gene expression of *hrpS* and *gacA* with the *p*>0.5, but gene expression of *hrpL, dspE, hrpA*, and *rsmB* are significantly different between MM and MM supplemented with 0.1 mM OCA with *p*<0.003.

Since OCA and TCA induced the expression of *hrpN*, we further investigated the effect of these two phenolic compounds on the expression of additional T3SS genes *hrpA* and *dspE* by qRT-PCR. Compared with MM alone, a significantly higher amount of *dspE* and *hrpA* mRNA was observed in Ech3937 supplemented with OCA (Fig. 3). As in previous work [31], the promoter activities of Ech3937 were determined by collecting the average GFP fluorescence intensity of total bacterial cells (Total) from a flow cytometry although three parameters were measured, including average GFP fluorescence intensity of total bacterial cells (Total), average GFP fluorescence intensity of GFP expressing bacterial cells (GFP^+^), and the percentage of GFP expressing bacterial cells of the total bacterial cells (GFP^+^%). Compared with MM alone, the average GFP fluorescence intensity of total bacterial cells (Total) of Ech3937 (phrpA) was doubled when 0.1 mM of OCA and TCA were added to the medium (Table 2). The *mrp*, whose protein product contains an ATPase conserved domain, was used as a reference gene in this study as in previous work [31]. Similar *mrp* expression was observed in Ech3937 (pmrp) when the bacterial cells were grown in MM and MM supplemented with 0.1 mM OCA and TCA, respectively (Table 2).

**Table 2:** The expression of *hrpA, hrpN, hrpL,* and *hrpS* of *Dickeya dadantii* 3937 (Ech3937) in MM, MM supplemented with 0.1 mM OCA (MMOCA), and MM supplemented with 0.1 mM TCA (MMTCA).

Gene Promoter^a^		MM	MM+OCA	MM+TCA
Ech3937 (p*hrpA*) b	Total	41.0±2.5	82.2±0.6	77.7±0.3
	GFP^+^	120.5±2.5	170.3±4.1	167.6±13.1
	GFP^+^%	31.0±1.8	46.5±1.1	44.8±3.5
Ech3937 (p*hrpN*)	Total	43.0±4.3	94.4±3.2	102.8±1.1
	GFP^+^	150.9±12.5	210.1±20.8	194.2±21.6
	GFP^+^%	27.1±3.1	44.2±4.6	52.6±6.3
Ech3937 (p*hrpL*)	Total	13.7±1.2	19.1±0.2	18.8±1.0
	GFP^+^	24.8±1.0	29.8±0.3	29.5±0.7
	GFP^+^%	41.7±4.2	54.9±0.8	54.3±3.3
Ech3937 (p*hrpS*)	Total	62.6±1.6	56.4±0.7	53.5±1.8
	GFP^+^	63.7±1.8	57.3±0.7	55.0±1.7
	GFP^+^%	98.1±0.3	98.2±0.1	97.0±0.3
Ech3937 (pAT)	Total	1.9±0.0	2.0±0.0	1.9±0.0
	GFP^+^	28.1±38.3	35.1±32.2	0
	GFP^+^%	0	0	0

a The promoter activities were compared at 12 h of grown in the media. GFP intensity was determined on gated populations of bacterial cells by flow cytometry. GFP intensity was determined on gated populations (10,000-15,000 events) of bacterial cells by flow cytometry. The fluorescence intensities were collected, including average GFP fluorescence intensity of total bacterial cells (Total), average GFP fluorescence intensity of GFP expressing bacterial cells (GFP^+^), and the percentage of GFP expressing bacterial cells of the total bacterial cells (GFP^+^%).

b Values (Mean Fluorescence Intensity) are a representative of at least 3 experiments with similar results. Three replicates were used in this experiment. The value is present as average of three replicates with standard deviation (SD).

### The effect of phenolic compounds at levels relevant in plants can induce T3SS

We analyzed whether the effect of phenolic compounds is at levels that are physiologically relevant in plants. The potato plant is one of the natural hosts of *D. dadantii*. Montesano et al. [29] reported that the concentration of the phenolic compound TCA in healthy potato leaves was approximately 0.5 µM, and that TCA accumulated to 10 µM in the leaves after exposure to cell-free culture filtrate (CF) of the phytopathogen *E. carotovora*. To investigate whether the level of the phenolic compounds in plants is able to induce the expression of the T3SS gene *hrpN*, we further examined its expression with concentrations of TCA equivalent to that in potato leaves. Ech3937 (phrpN) was grown in MM supplemented with 0.2, 0.5, 5 and 10 µM of TCA, respectively. Compared with Ech3937 (phrpN) in MM alone, a 1.5- to 1.8-fold increase of GFP intensity was observed in the bacterial cells grown in MM supplemented with 0.2 and 0.5 µM TCA (Table 3). Compared with Ech3937 (phrpN) in MM, a 3- to 3.5-fold higher GFP intensity was observed in the bacterial cells grown in MM supplemented with 5 and 10 µM of TCA (Table 3). These observations suggest that physiologically relevant concentrations of phenolic acids can induce *hrpN*.

**Table 3:** The expression of *hrpN* of *Dickeya dadantii* 3937 (Ech3937) in MM and MM supplemented with different amount of TCA and SA respectively.

		12 h	24 h
**MM** a	Total^b^	41.6±3.6	91.3±11.5
	GFP^+^	196.8±6.6	194.5±14.3
	GFP^+^%	20.0±2.3	46.1±7.3
**TCA** a			
0.2 µM	Total	65.1±6.9	163.1±25.5
	GFP^+^	200.0±11.4	250.7±20.2
	GFP^+^%	31.5±3.2	64.2±5.4
0.5 µM	Total	73.5±4.3	158.8±21.0
	GFP^+^	228.0±8.9	251.4±18.0
	GFP^+^%	31.4±3.2	62.6±6.7
5 µM	Total	134.7±5.7	266.1±14.5
	GFP^+^	287.9±24.0	357.4±9.1
	GFP^+^%	46.3±2.2	74.1±2.9
10 µM	Total	147.8±18.5	284.3±12.3
	GFP^+^	304.1±26.7	397.7±7.7
	GFP^+^%	48.0±3.2	71.1±3.4
**SA** a			
0.2 µM	Total	53.8±4.0	105.6±28.7
	GFP^+^	199.6±3.1	219.3±7.0
	GFP^+^%	25.9±2.5	47.0±11.8
0.5 µM	Total	49.6±6.0	99.6±18.8
	GFP^+^	205.0±2.6	190.9±14.5
	GFP^+^%	23.1±3.3	50.9±6.1
5 µM	Total	50.0±3.0	97.7±9.0
	GFP^+^	200.2±1.6	194.0±1.0
	GFP^+^%	23.8±1.3	49.4±4.5
10 µM	Total	52.1±2.4	104.7±3.2
	GFP^+^	217.4±5.0	196.2±7.4
	GFP^+^%	22.9±0.7	52.5±3.1

a Minimal medium (MM) alone and MM supplemented with different concentrations of *t*-cinnamic acid (TCA) or salicylic acid (SA).

b The promoter activities of *hrpN* were measured at 12 and 24 h of growing in the media. The fluorescence intensities were collected, including average GFP fluorescence intensity of total bacterial cells (Total), average GFP fluorescence intensity of GFP expressing bacterial cells (GFP^+^), and the percentage of GFP expressing bacterial cells of the total bacterial cells (GFP^+^%). Three replicates were used in this experiment. The value (Mean Fluorescence Intensity) is present as the average of three replicates with standard deviation. The GFP intensities of Ech3937 (pAT) grown in MM were 2.2±0 and 3.4±0.1 at 12h and 24h respectively.

### Effect of OCA on IAA biosynthesis pathway

Since an induction of the expression of *hrpA, hrpN* and *dspE* was observed in Ech3937, the regulatory mechanism of these phenolic compounds on the T3SS pathway was investigated. Our previous work demonstrated that the expression of T3SS genes *dspE, hrpA*, and *hrpN* was reduced in an *iaaM* mutant Ech138; *iaaM* encodes an enzyme in the pathway for indole-3-acetic acid (IAA) biosynthesis [32]. To investigate whether IAA biosynthesis is involved in induction of T3SS by the phenolic compounds, the expression of *hrpN* in the wild-type Ech3937 and Ech138 was compared with the addition of OCA. As expected, the expression of *hrpN* was reduced in an *iaaM* mutant background. However, a similar induction ratio of *hrpN* by OCA was observed in wild-type Ech3937 and Ech138 at each time point of bacterial growth (Table 4). These results suggest that OCA does not induce T3SS expression through IAA biosynthesis pathway.

**Table 4:** The expression of *hrpA* or *hrpN* of *Dickeya dadantii* 3937 (Ech3937), *gacA* mutant Ech137, *iaaM* mutant Ech138, *rsmB* mutant 148 and *gacS* mutant Ech149 in MM and MM supplemented with 0.1 mM OCA (MMOCA).

Gene Promoter^a^		6h		12 h		24 h	
		MM	MMOCA	MM	MMOCA	MM	MMOCA
Ech3937 (pAT) b	Total	1.9±0.0	2.0±0.0	1.9±0.0	2.0±0.0	1.8±0.0	1.9±0.0
	GFP^+^	3.9±6.7	8.0±7.1	28.1±38.3	35.1±32.2	16.3±7.2	10.7±0.0
	GFP^+^%	0	0	0	0	0	0
Ech-Rif (phrpA)	Total	12.7±0.2	17.5±0.2	22.1±0.4	51.3±1.5	16.1±1.2	49.2±0.8
	GFP^+^	100.8±4.5	106.7±4.8	111.2±1.7	142.8±2.1	67.1±3.7	97.7±2.4
	GFP^+^%	8.1±0.5	12.0±0.3	16.2±0.2	33.4±0.5	19.7±2.5	48.0±0.3
Ech137 (phrpA)	Total	4.7±0.0	4.9±0.1	5.5±0.0	5.9±0.1	7.6±0.0	8.3±0.0
	GFP^+^	19.3±4.2	18.3±3.4	14.9±0.9	15.4±0.3	14.0±0.0	14.3±0.3
	GFP^+^%	2.2±0.1	2.4±0.1	4.3±0.3	6.2±0.4	18.4±0.3	23.3±0.3
Ech3937 (phrpN)	Total	18.8±2.4	31.4±3.2	29.5±3.1	91.4±8.0	32.5±2.0	78.1±7.9
	GFP^+^	151.3±9.8	171.7±10.8	149.2±8.6	232.6±36.2	98.9±6.3	162.4±11.4
	GFP^+^%	10.4±2.1	16.4±2.5	17.7±1.3	38.5±3.2	30.9±3.2	46.9±3.5
Ech138 (phrpN)	Total	5.6±0.7	9.1±1.6	11.3±0.9	32.6±5.0	17.3±1.5	38.7±5.3
	GFP^+^	68.6±8.4	69.6±6.7	95.2±7.0	115.3±4.5	90.5±1.1	105.8±7.5
	GFP^+^%	3.3±0.6	8.2±1.6	8.4±0.3	25.7±5.4	16.3±1.8	34.5±2.6
Ech148 (phrpN)	Total	4.3±0.1	3.8±0	4.2±0.1	3.9±0.1	4.8±0.2	4.8±0.1
	GFP^+^	16.7±3.2	15.2±0.9	22.9±9.2	27.8±7.5	22.8±6.4	23.6±6.1
	GFP^+^%	0.6±0.1	0.4±0	1.6±0.3	0.8±0.2	2.69±1.0	2.3±0.4
Ech149 (phrpN)	Total	4.6±0.2	3.6±0.3	4.7±0.3	3.9±0.2	4.6±0.2	5.4±0.2
	GFP^+^	14.8±1.3	26.7±15.4	17.1±1.3	36.0±13.4	32.6±12.2	37.8±12.5
	GFP^+^%	0.6±0.3	0.3±0	2.6±1.2	0.9±0.2	2.2±0.1	2.9±0.1

a The promoter activities were compared at 6, 12, and 24 h of bacterial growth. The fluorescence intensities were collected, including average GFP fluorescence intensity of total bacterial cells (Total), average GFP fluorescence intensity of GFP expressing bacterial cells (GFP^+^), and the percentage of GFP expressing bacterial cells of the total bacterial cells (GFP^+^%).

b Values are a representative of at least two experiments with similar results. Three replicates were used in this experiment. The value is present as the average of three replicates with standard deviation (SD).

### Effect of OCA on HrpX/HrpY-HrpS-HrpL pathway

To investigate whether the OCA induces the T3SS through HrpX/HrpY-HrpS-HrpL pathway, the promoter activities and mRNA levels of *hrpS* and *hrpL* of 3937 was examined in MM and MM supplemented with 0.1 mM OCA. Similar *hrpS* promoter activities and *hrpS* mRNA levels were observed between bacterial cells grown in MM and MM supplemented with OCA or TCA (Table 2 and Fig. 3). Compared with MM alone, a slightly higher promoter activity of *hrpL* was observed in Ech3937 (*hrpL*) grown in MM supplemented with OCA and TCA (Table 2). However, Ech3937 cultures with the supplementation of 0.1mM OCA produced about 3-fold more *hrpL* mRNAs than those grown in MM alone at 12 h of growth (*p*<0.01) (Fig. 3). These results suggest that OCA does not activate T3SS expression through HrpX/Y-HrpS-HrpL pathway and these phenolic compounds induce *hrpL* expression at a post-transcriptional level.

### *rsmB* up-regulates *hrpL* gene expression at a post-transcriptional level

**Figure 4:**
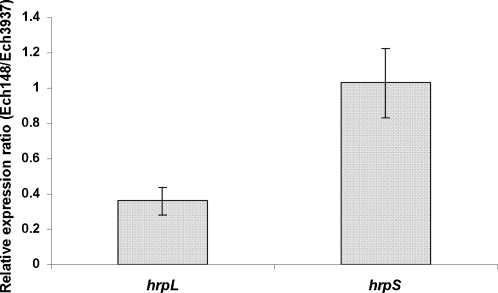
The relative level of mRNA of *hrpL* and *hrpS* in *Dickeya dadantii* 3937 (Ech3937) and *rsmB* mutant Ech148 grown for 12 h in minimal medium. The amount of mRNA was determined by qRT-PCR. Three replicates were used in this experiment. The *p*-value was calculated using Relative Expression Software Tool as described by Pfaffl et al. [41]. There is no significant difference between Ech3937 and Ech148 for gene *hrpS* with the *p*>0.3, but gene expression of *hrpL* is significantly different between Ech3937 and Ech148 with *p*<0.008.

The pPROBE-AT is a promoter-probe reporter plasmid [33], [34]. Since the *gfp* of pPROBE-AT contained its own ribosome binding site, promoter activities of bacterial cells were measured when a promoter region was inserted into this vector [31]. In *E. carotovora*, RsmA-*rsmB* regulated *hrpL* expression at the post-transcriptional level [15], [17]. In this study, a *rsmB* mutant Ech148 was constructed, and a reduced amount of *hrpL* mRNA was observed in this mutant in comparison with 3937 (Fig. 4). However, similar promoter activity of *hrpL* was observed between the wild-type bacterium and Δ*rsmB* mutant Ech148 (Table 5). Similar promoter activity and mRNA level of *hrpS* were observed between 3937 and Ech148 mutant. These results suggested that the reduced amount of *hrpL* mRNA in Ech148 was due to the lack of *rsmB* RNA to quench the activity of RsmA in Ech148. In addition, compared to Ech3937, a lower expression of downstream T3SS genes *hrpA* and *hrpN* was observed in mutant Ech148 (Table 5), which was due to a reduced amount of *hrpL* mRNA in this mutant (Fig. 4).

**Table 5:** The promoter activities of *hrpS, hrpL, hrpA* and *hrpN* of *Dickeya dadantii* 3937 (Ech3937) and *rsmB* mutant Ech148 grown in minimum medium.

Gene Promoter^a^		6h	12 h
Ech3937 (pAT) b	Total	2.1±0.1	2.9±0.6
	GFP^+^	12.6±1.6	29.9±10.3
	GFP^+^%	0	0
Ech3937 (phrpS)	Total	35.9±1.1	107.4±0.9
	GFP^+^	39.6±0.9	108.0±1.1
	GFP^+^%	88.0±1.1	99.5±0.2
Ech148 (phrpS)	Total	30.6±2.0	90.6±3.3
	GFP^+^	33.1±1.9	90.9±3.4
	GFP^+^%	90.4±1.8	99.8±0.1
Ech3937 (phrpL)	Total	4.7±0.2	10.0±1.1
	GFP^+^	16.3±1.2	16.5±2.2
	GFP^+^%	3.3±0.4	34.1±3.2
Ech148 (phrpL)	Total	5.3±0.0	9.0±0.1
	GFP^+^	14.3±0.5	14.1±0.1
	GFP^+^%	3.4±0.2	29.9±1.1
Ech3937 (phrpA)	Total	7.6±0.1	90.7±6.5
	GFP^+^	58.0±1.7	176.4±9.2
	GFP^+^%	5.6±0.2	49.8±4.8
Ech148 (phrpA)	Total	4.7±0.1	8.5±0.1
	GFP^+^	16.5±0.5	16.0±0.2
	GFP^+^%	1.5±0.2	21.6±0.9
Ech3937 (phrpN)	Total	4.2±0.3	63.7±3.4
	GFP^+^	79.0±4.7	231.4±33.0
	GFP^+^%	1.9±0.3	26.6±2.5
Ech148 (phrpN)	Total	2.5±0.1	3.84±0.1
	GFP^+^	35.3±6.2	30.3±1.8
	GFP^+^%	0.3±0.1	1.4±0.2

a The promoter activities were compared at 6 and 12 h of bacterial growth. The fluorescence intensities were collected, including average GFP fluorescence intensity of total bacterial cells (Total), average GFP fluorescence intensity of GFP expressing bacterial cells (GFP^+^), and the percentage of GFP expressing bacterial cells of the total bacterial cells (GFP^+^%).

b Values are a representative of three experiments with similar results. Three replicates were used in this experiment. The value is present as the average of three replicates with standard deviation (SD).

### OCA induces T3SS through *rsmB*-RsmA pathway

The TCS GacS/GacA up-regulates *rsmB* and RsmA-*rsmB* regulates *hrpL* at the post-transcriptional level (Fig. 1). To investigate if OCA induces the T3SS through the RsmA-*rsmB* pathway, the promoter activities of *hrpA* or *hrpN* were compared in the wild-type bacterium, Δ*rsmB* mutant Ech148, Δ*gacS* mutant Ech149 and Δ*gacA* mutant Ech137 carrying phrpA or phrpN grown in MM and MM supplemented with OCA respectively. The wild-type showed a higher GFP intensity grown in MM supplemented with OCA in comparison to MM alone. However, similar GFP intensity was observed in Ech137, Ech148 and Ech149 cells grown in MM and MM supplemented with OCA at each time point of bacterial growth (Table 4). The effect of OCA on the expression of *gacA* and *rsmB* was further examined by qRT-PCR. Our results show that, compared with Ech3937 in MM alone (normalized to 1), a significantly higher *rsmB* mRNA (relative expression ratio 1.4, *p* = 0.003) was observed in the bacterium grown in MM supplemented with OCA (Fig. 3). However, no significant difference in the level of *gacA* mRNA was observed in Ech3937 grown in MM and MM supplemented with OCA (Fig. 3). These results suggest the OCA and TCA induce the T3SS through the *rsmB*-RsmA pathway.

## Discussion

Plants have multifaceted strategies to deal with microbial pathogens by producing a wide array of antimicrobial compounds, such as phenolic compounds [26]. In the SA biosynthesis pathway in plants, TCA is converted to OCA through *ortho*-hydroxylation. SA is produced by β–oxidation of OCA [35]. An increase of the phenolic acid TCA was observed in potato leaves at 2 h after exposure to CF from *E. carotovorum* [29]. In addition, TCA was shown to induce the expression of defense-related genes *drd-1* (a defense-related alcohol dehydrogenase), *pinII* (proteinase inhibitor II), *chtB4* (basic chitinase) and *chtA2* (acidic chitinase) of potato, suggesting that TCA may play a role in defense signaling in plants. The T3SS is considered one of the major virulence factors in many bacterial pathogens. T3SS delivers effectors into host cells [36]. One major role of T3SS of phytopathogens is to disable the host defense system during bacterial invasion. In this work, an approximately 1.7-fold higher GFP intensity was observed in Ech3937 (p*hrpN*) grown in MM supplemented with 0.5 µM of TCA (roughly the level of TCA in potato leaves) in comparison to bacterial cells grown in MM alone (Table 3). However, an approximately 3-fold higher GFP intensity was observed in the bacterial cells grown in MM supplemented with 5 µM of TCA (the level of TCA in potato leaves induced by CF). This result indicates that Ech3937 may modulate its T3SS expression to invade hosts by sensing the basal level of TCA in healthy host plants. In addition, due to the accumulated level of the phenolic compound in hosts caused by bacterial infection, a higher expression of T3SS may be induced in the bacterial cells for a defensive response against the plant responses.

In this study, a higher amount of mRNAs of *dspE* and *hrpA* was observed when OCA or TCA were added into MM (Fig. 3). No induction was observed in *hrpS* when these two plant phenolic acids were added to the MM. Since a significant increase of mRNA of *hrpL* but only a slight increase of the *hrpL* promoter activity of Ech3937 was observed when these two plant phenolic acids were added into the MM (Table 3; Fig. 3), it is plausible that *dspE, hrpA* and *hrpN* are induced by an alternative regulatory pathway and not the HrpX/HrpY-HrpS pathway. In this study, OCA and TCA were unable to enhance *hrpA* or *hrpN* expression of the Δ*rsmB,* Δ*gacS, and* Δ*gacA* mutants of Ech3937 (Table 4). In addition, an increased mRNA level of *rsmB* in the wild-type Ech3937 was observed when OCA and TCA were added in MM (Fig. 3). These results suggest that these phenolic compounds regulate T3SS through *rsmB*-RsmA pathway. Since the expression of *rsmB* of Ech3937 is up-regulated by TCS GacS/GacA [15], our results suggest that OCA/TCA may induce the T3SS gene expression by modulating the mRNA level of *rsmB* through activation of GacS/GacA. Compared with MM alone, there was no increase in *gacA* mRNA of Ech3937 when the bacterial cells were grown in MM supplemented with OCA (Fig. 3). In TCS, the activity of histidine kinase and response regulator is stimulated by the phosphorylation of histidine and aspartate residues of these TCS proteins respectively [37]. In this work, the activation of TCS GacS/GacA by OCA may result from the phosphorylation of the GacA protein through GacS. Thus, the amount of mRNA of *gacA* may not be increased when OCA is supplemented in MM. In the *envZ-ompR* TCS, the role of EnvZ is primarily as a phosphodonor for response regulator OmpR activation [38]. Disruption of *envZ*, the sensor kinase did not reduce the level of mRNA of the response regulator *ompR*. However, at this stage, we can not rule out the possibility of other unknown regulator(s) affected by OCA and further up-regulating the expression of *rsmB*. Finally, compared with Ech3937 in MM, a slightly higher promoter activity of *hrpL* was observed in the bacterial cells grown in MM supplemented with OCA. With the complexity of the T3SS regulatory system revealed in Ech3937, we can not rule out that other alternate regulatory pathways may also play a role in T3SS induction by OCA and/or TCA.

In summary, two T3SS inducers, OCA and TCA, were identified in this study. The induction of T3SS gene expression by these two phenolic compounds is moderated through the *rsmB*-RsmA pathway. With the similarity of these global virulence regulatory systems of T3SS among plant and animal pathogens, the roles of plant phenolic compounds on Ech3937 unveiled in this study will foster efforts for the future development of antimicrobial reagents (e. g., development of phenolic compound analogues that block the T3SS regulatory pathway) and strategies for pathogen control in many fields, including agriculture, medicine, and the food industry.

## Materials and Methods

### Bacterial strains, plasmids, media and chemicals

The bacterial strains and plasmids used in this study are listed in Table 1. *E. coli* was grown in LB broth at 37°C and *D. dadantii* was grown in MM at 28°C [32]. Antibiotics (µg/ml) used were: ampicillin, 100; kanamycin, 50. Primers used for Polymerase Chain Reaction (PCR) in this report are also listed in Table 1. OCA, TCA and SA were purchased from Sigma-aldrich (St. Louis, MO). A transposon *miniHimar RB1* was used to construct a mutant library of 3937 in the Yang Lab (unpublished data). For this purpose, *E. coli* S17-1 λ-pir (pMiniHimar RB1) (*E. coli* S17-1 λ-pir cells carrying plasmid pMiniHimar RB1) was used as a donor in mating with Ech3937 [39]. The mini*Himar RB1* carries an R6Kγ origin of replication. To locate the disrupted region containing the Mini*Himar RB1*, the chromosomal DNAs of these mutants were digested by *Bam*H1, followed by self-ligation and sequencing [39]. Two of the transposon mutants, Δ*rsmB* (Ech148) and Δ*gacS* (Ech149), identified in the mutant library were used in this study.

### FACS analysis

FACS analysis of promoter activity of *hrpA, hrpL, hrpN,* and *hrpS* was carried out as described [31]. Briefly, the wild-type Ech3937 and the mutant strains carrying the promoter reporter plasmid were grown on LB broth at 28°C overnight and transferred to appropriate media. For FACS analysis, samples were collected by centrifugation, washed with 1X phosphate buffer saline, and re-suspended in 1X PBS to ca 10^6^ CFU/ml prior to being run in a FACS Calibur flow cytometer (BD Biosiences, CA). Among all the flow cytometry assays that we tested, the gated event number of each individual assay was constantly around 10000–15000 events. To avoid debris, electronic background, and undesired clumps in the bacterial samples, a Gate R1 was set up, which was based on light scatter for the flow cytometry assay.

### qRT-PCR analysis

Bacterial strains were grown in MM. Total RNA from the bacterial cells was isolated by using the TRI reagent method (Sigma, MO) and treated with Turbo DNA-free DNase kits (Ambion, TX) as described [31]. An iScript cDNA Synthesis Kit (Bio-Rad, Hercules, CA) was used to synthesize cDNA from 0.5 µg of treated total RNA. The Real Master Mix (Eppendorf, Westbury, NY) was used for qRT-PCR reactions to quantify the cDNA level of target genes in different samples. The *rplU* was used as the endogenous control for data analysis [40]. qRT-PCR data were analyzed using Relative Expression Software Tool as described by Pfaffl et al. [41].

## Supporting Information

Figure S1 The growth of Ech3937 (phrpN) in MM and MM supplemented with different concentrations of OCA, TCA and SA. Overnight-cultured Ech3937 (phrpN) cells in Luria Broth were transferred into MM or MM supplemented with OCA, TCA and SA. At 12h and 24h, serial dilutions of bacterial cultures were plated on the Luria Broth agar. The Colony Forming Unit (CFU) per ml was obtained according to the numbers of the colonies growing on the plates at different dilutions. Three replicates were used in this experiment.
(0.02 MB TIF)
