# Correction: Type III Secretion System Genes of *Dickeya dadantii* 3937 Are Induced by Plant Phenolic Acids

**DOI:** 10.1371/annotation/91170966-226f-4678-999e-22f2c4a6bb8d

**Published:** 2008-09-26

**Authors:** Shihui Yang, Quan Peng, Michael San Francisco, Yongjun Wang, Quan Zeng, Ching-Hong Yang

The first two authors, Shihui Yang and Quan Peng, should be noted as contributing equally to this work.

